# Low quality of maternal and child nutritional care at the primary care in Mexico: an urgent call to action for policymakers and stakeholders

**DOI:** 10.1186/s12939-024-02129-z

**Published:** 2024-02-22

**Authors:** Omar Acosta Ruiz, Monica Ancira-Moreno, Isabel Omaña-Guzmán, Sonia Hernández Cordero, Arturo Cuauhtémoc Bautista Morales, Cecilia Pérez Navarro, Soraya Burrola Méndez, Eric Monterrubio Flores, Alejandra Trejo, Martha Kaufer-Horwitz, Ariana Cajero, Belén Sánchez, Constanza Bernat, Elder Salgado-Amador, Elizabeth Hoyos-Loya, Mónica Mazariegos, Cinthya Muñoz Manrique, Royer Pacheco Cruz, Elvia Mendoza, Mauro Brero, Matthias Sachse, Fernanda Cobo Armijo

**Affiliations:** 1 Center for Research in Evaluation and Surveys, National Institute of Public Health, Cuernavaca, Mexico; 2 Health Department, Universidad Iberoamericana, Mexico City, Mexico; 3 Observatorio Materno Infantil (OMI), Universidad Iberoamericana, Mexico City, Mexico; 4 Pediatric Obesity Clinic and Wellness Unit, Hospital General de México, “Dr. Eduardo Liceaga,”, Mexico City, Mexico; 5 Research Center for Equitable Development EQUIDE, Universidad Iberoamericana, Mexico City, Mexico; 6 Center for Nutrition and Health Research, National Institute of Public Health, Cuernavaca, Mexico; 7 Dirección de Nutrición, Instituto Nacional de Ciencias Médicas y Nutrición Salvador Zubirán, Mexico City, Mexico; 8 Research Center for the Prevention of Chronic Diseases (CIIPEC), Institute of Nutrition of Central America and Panama (INCAP), Guatemala City, Guatemala; 9 Departamento de Nutrición y Bioprogramación, Instituto Nacional de Perinatología, Mexico City, Mexico; 10 Instituto de Nutrición, Universidad de la Sierra Sur, Oaxaca, México; 11 United Nations International Children’s Emergency Fund (UNICEF), Mexico City, México

**Keywords:** Quality care, Nutritional care, Primary health care, Maternal nutrition, Child nutrition, Quality care indicators, Mexico, Calidad de atención, Atención nutricional, Atención primaria de salud, Nutrición materna, Nutrición infantil, Indicadores de calidad de atención, México

## Abstract

**Background:**

Maternal and child malnutrition represents a public health problem in Mexico Primary care (PC) is responsible for introducing women and children under five to the health system, detecting diseases on time, and providing medical services, including pharmacological treatment if necessary. Providing these services with quality is essential to improve maternal and child health. This study evaluated the quality of nutritional care during preconception, pregnancy, postpartum, infancy, and preschool age at the PC health units across six Mexican states between 2020 and 2021.

**Methods:**

We conducted a cross-sectional study with a mixed approach in units of the Secretary of Health to assess the quality of nutritional care during preconception, pregnancy, postpartum, childhood, and preschool age. The level of quality was calculated by the percentage of compliance with 16 indicators that integrated a Quality Index of Maternal and Child Nutritional Care (ICANMI, by its Spanish acronym). Compliance by indicator, by life stage, and overall was categorized using the following cut-off points: poor quality (≤ 70%), insufficient quality (71-89%), and good quality (≥ 90%). The perceptions of the barriers and facilitators that affect maternal and child nutrition were evaluated through semi-structured interviews with health professionals (HP) and users. All qualitative instruments were developed with a gender and intercultural perspective.

**Results:**

Considering the whole sample studied, maternal and child nutritional care quality during the five life stages evaluated was bad (compliance: ≤12%), reflected in the ICANMI, which had a compliance of 8.3%. Principal barriers identified to providing high-quality nutritional care were the lack of knowledge and training of health professionals, shortages of equipment, medicine, personnel, and materials, the disappearance of the social cash transfer program Prospera, the absence of local indigenous language translators to support communication between doctor and patient, and the persistence of machismo and other practices of control over women.

**Conclusions:**

These findings underscore the need for initiatives to improve the quality of nutritional care in PC facilities across Chihuahua, State of Mexico, Veracruz, Oaxaca, Chiapas, and Yucatan. It is necessary for government and health authorities, along with various stakeholders, to collaboratively devise, implement, and assess intercultural and gender-oriented policies and programs geared towards ensuring the health infrastructure and enhancing the training of health professionals to diagnose and treat the prevalence and occurrence of diverse forms of malnutrition in both maternal and child populations.

**Supplementary Information:**

The online version contains supplementary material available at 10.1186/s12939-024-02129-z.

## Resumen

**Antecedentes:**

La mala nutrición materno-infantil (MMI) representa un problema de salud pública en México. El primer nivel tiene la respondabilidad de introducir a mujeres y niños menores de 5 años al sistema de salud, detectar oportunamente las enfermedades y brindar servicios médicos incluido el farmacológico de ser necesario. Prestar estos servicios con calidad resulta elemental para mejorar la salud de la población materno-infantil. El objetivo de este estudio fue evaluar la calidad de la atención nutricional durante las etapas de preconcepción, embarazo, posparto, infancia y edad preescolar en centros de salud de seis estados de México entre 2020 y 2021.

**Métodos:**

Se realizó un estudio transversal con metodología mixta en 95 centros de salud la Secretaría de Salud de México para evaluar la calidad de la atención nutricional durante la preconcepción, el embarazo, el posparto, la infancia y la etapa preescolar. El nivel de calidad se calculó mediante el porcentaje de cumplimiento de 16 indicadores que a su vez integraron un Índice de Calidad de la Atención Nutricional Materno Infantil (ICANMI). El cumplimiento por indicador, etapa de vida y global fue categorizado utilizando los siguientes puntos de corte: mala calidad (≤ 70%), calidad insuficiente (71-89%) y buena calidad (≥ 90%). La percepción sobre las barreras y facilitadores que afectan la atención nutricional materno-infantil fueron identificadas a través de entrevistas semiestructuradas y grupos focales realizadas a profesionales de salud, usuarias y usuarios. Todos los instrumentos cualitativos fueron desarrollados con un enfoque de género e interculturalidad.

**Resultados:**

La calidad de la atención nutricional materno infantil durante las cinco etapas de la vida evaluadas fue mala (cumplimiento: ≤12%), mientras que el ICANMI tuvo un cumplimiento de 8.3%. Las principales barreras identificadas para brindar una atención nutricional de alta calidad fueron la falta de conocimiento y capacitación de los profesionales de la salud, la escasez de equipos, medicamentos, personal y materiales, la desaparición del programa social de transferencias monetarias Prospera, la ausencia de una lengua indígena local, entre otros. Así como la persistencia de prácticas como el machismo y otras de control sobre las mujeres.

**Conclusiones:**

Estos hallazgos subrayan la necesidad inmediata de implementar iniciativas que mejoren el estándar de atención nutricional en los centros de salud en Chihuahua, Estado de México, Veracruz, Oaxaca, Chiapas y Yucatán. Es necesario que el gobierno y las autoridades sanitarias, junto con diversas partes interesadas, diseñen, implementen y evalúen en colaboración políticas y programas orientados a mejorar la calidad de la atención nutricional, con perspectiva de género e interculturalidad. Este esfuerzo tiene como objetivo mitigar la prevalencia y aparición de diversas formas de desnutrición tanto en la población materna como infantil.

## Background

In the current epidemiological context, maternal and child malnutrition represents a global public health problem [1]. In 2019, 2.94 million deaths were attributable to child and maternal malnutrition worldwide [2]. In Mexico, in 2022, the prevalence of stunting in children under five years of age was 12.8%, while for underweight, the prevalence was 4.1%; for wasting, it was 0.8%; and for overweight, it was 7.7% [3]. In 2017, the national incidence of low birth weight was 7.1%, with a progressive increase since 2008, when the incidence was 6.2% [4]. Moreover, the prevalence of overweight and obesity in women of reproductive age was 76% in 2020 [5].

Maternal and child malnutrition, either due to deficiency or excess, is associated with adverse consequences on the health of the mother-child binomial in the short and long term. The first 1,000 days of life are a critical period of growth and development during which malnutrition may have irreversible adverse consequences on the future health of individuals. However, this period of life also represents a window of opportunity to establish interventions to improve children’s growth and development [6, 7].

The evidence points out that maternal health and nutrition status before conception and during pregnancy [8] play a crucial role in pregnancy, childbirth, and the health of the offspring [8, 9]. Both maternal underweight and obesity are associated with an increased risk of maternal complications during pregnancy [10] and adverse effects on fetal growth [11, 12]. On the other hand, it is estimated that more than half of pregnancy-related deaths occur during the postpartum [13].

In Mexico, the Ministry of Health (SS) and its 32 State Health Secretariats (SESA) and IMSS-Bienestar provide services at primary and secondary care to the population without social security (non-salaried workers, such as farmers, small merchants, and professionals in the independent exercise of their activities, as well as the unemployed and people outside the labor market, such as housewives and their families. In 2020, this population represented 36.5% of all habitants, 33,801,552 Mexicans [14, 15]. Consisting mainly of health centers, the PC has an essential core of physicians, nurses, and health promoters and provides essential health services [16]. Its responsibility is to introduce the population to the health system, detect malnutrition promptly, provide medical services, including pharmacological treatment if necessary, and provide control of pregnancy and children under five years of age, as well as other services.

According to the World Health Organization (WHO) [17], high-quality nutritional care is a critical component of Primary Care (PC). Therefore, it is essential to ensure an adequate quality of nutritional care during preconception, pregnancy, postpartum, and childhood to prevent, diagnose, and treat all forms of malnutrition during these critical stages of life and diminish the adverse effects on mothers and their newborns in the short and long term [18].

Even though maternal and child malnutrition represents a public health problem with crucial short-term health consequences and considering the importance of PC for its prevention and management, we did not find a study that evaluated the quality of nutritional care in this population at PC.

The general objective of this study was to evaluate the quality of nutritional care during preconception, pregnancy, postpartum, infancy, and preschool age stages at the PC health units across six Mexican states between 2020 and 2021. To achieve this, two specific objectives were included: (1) To assess the quality of the nutrition care process for preventing, diagnosing, and treating malnutrition during the mentioned life stages, and (2) To identify health personnel’s (HP) knowledge regarding quality care, nutritional information, and recommendations applicable during the care process, as well as the perceived barriers and facilitators they encounter when interacting with users.

## Methods

### Study design

We carried out a cross-sectional and observational study with a mixed approach in health units of six Mexican states located in the center (State of Mexico), east (Veracruz), southeast (Oaxaca, Chiapas, and Yucatan), and north (Chihuahua) regions of the country from September to December 2021. The states were selected intentionally in collaboration with the Mexican Ministry of Health, ensuring the inclusion of at least one state from each geographic region. Additionally, states with a significant proportion of indigenous populations and high poverty rates were included. Oaxaca, Chiapas, and Yucatan have the highest number of indigenous language speakers in the country, with a proportion of 31.2, 28.2 and 23.7%, respectively; Chihuahua has the highest proportion in the north of the country, with 3.1%; and the states of Mexico and Veracruz have 8.6% and 2.6%, respectively [19]. Chiapas, Oaxaca, Veracruz, and the State of Mexico are among the top 10 states of poverty nationwide [20].

A sample of 97 health units was selected, considering the total number of health centers (*n* = 4,121) in the six selected states, expecting a percentage of 50% of medical units with a good level of quality of care, a 95% confidence level and a 10% margin of error. The selection of the medical units was carried out using stratified simple random sampling; the following four stratums were considered for each state: (1) health units located in non-indigenous rural areas; (2) health units located in non-indigenous urban areas; (3) health units located in indigenous rural areas; and (4) health units located in indigenous urban areas (Supplementary Table 1).

The National Center for Child and Adolescent Health (CeNSIA, per its acronym in Spanish) provided all information related to the health centers. The fieldwork was conducted by ten trained researchers (five nutritionists, two psychologists, an anthropologist, a physician, and a nurse) and a general project manager.

### Quantitative methods

We developed and validated 16 composed indicators constructed upon 32 sub-indicators to evaluate the quality of nutritional care in five stages of maternal and child life. Of the total indicators, eight correspond to the group of women in the reproductive stage (preconception (2), pregnancy (4), and postpartum (2)); 5 indicators in childhood (0 to 24 months); and 3 in preschoolers (2–5 years) (Supplementary Table 2). The design and validation methodology of the 16 used indicators has been previously published [21].

The sources of information to assess the quality indicators were the clinical records. A random sample of 30 clinical records for each life stage (preconception, pregnancy, and postpartum; infancy and preschool age) was selected in each evaluated health unit. The sample size was chosen with a minimum requirement of 30 clinical records to identify quality issues, prioritizing the feasibility of measurement in a long-term monitoring plan. In health units where the total number of clinical records was less than 30, all available records were reviewed [22]. Only nine units did not reach the sample size in the evaluated stages. Only records of the population that attended health services from January 2020 to December 2021 were included.

The All-or-None approach was applied to evaluate the percentage of compliance of each indicator [23, 24]. This method considers that compliance with a composite indicator corresponds to the compliance rate in the cases where there was compliance with all the sub-indicators that comprise it [24].

The Lot Quality Acceptance Sampling (LQAS) method Campo [22] was used to analyze the health units’ level of quality. This method is based on a binomial distribution applicable to small sample sizes. This methodology’s modified American National Standards Institute (ANSI) tables were consulted, considering an error of α ≤ 0.05 and β ≤ 0.10 to identify the compliance percentage’s cut-off points. The quality level was classified as bad if compliance with the indicator was ≤ 70, poor quality between 71 and 89%, and good quality when compliance was ≥ 90%.

A quality index was generated for each life stage by averaging compliance with the indicators in each one of the stages, by health unit, and by state. This way, five indices were obtained (preconception, pregnancy, postpartum, infancy, and preschool index). In addition, a Quality Index of Maternal and Child Nutrition Care (ICANMI) was constructed considering the five life stages based on the average percentage of compliance with the 16 indicators that composed it [21]. These results were averaged to obtain the index by a federal entity for each of the life stages. The maternal and child nutritional care quality index was defined from the average of these indices for each health unit and a state level.

A traffic light system was used to present the results. According to the quality level mentioned before, green was used for good quality, yellow for poor quality, and red for bad quality. The units in which no evidence of records was found were classified as 0% compliance under the assumption that the activities needed to be carried out; this suggests problems in quality care.

### Qualitative methods

Interviews with key informants and focus groups were conducted as information-gathering techniques. The key informants were (a) women in the preconception stage, (b) pregnant women, (c) mothers, fathers, or caregivers of children under five, and (d) health providers who worked in the units during the data collection. It was necessary for all participants to be at least 18 years of age and to provide signed informed consent to participate in the research.

Given the characteristics of the target population, eight specific semi-structured interview guides were created for (1) physicians, (2) nurses, (3) nutritionists, (4) women in the preconception stage, (5) pregnant women, (6) women in the postpartum period, (7) mothers, fathers, or caregivers of babies from 0 to 2 years old, and (8) mothers, fathers or caregivers of children from 3 to 5 years.

These instruments aimed to identify the knowledge of health personnel about quality care, the nutritional information and recommendations they provide to users, and the barriers and facilitators they perceive to affect the care they provide. Regarding users (pregnant women, women in the pre-pregnancy period, women or men who attended the health unit with their daughters or sons), we sought to investigate the quality of the care they receive and their perception of the concept of quality of care. The above is from a perspective of gender and interculturality.

The instruments were developed under the phenomenological approach [25], including a series of general or contextual questions and specific batteries on prevention, diagnosis, and treatment, depending on the interviewee, that is, the type of health personnel or the stage of life in which the user was. In the case of health personnel, the questions focused on the facilitators and barriers to providing quality care and their perception of what this concept means. For the users, the questions sought to delve deeper into the experience of the care they receive from health personnel and their perception of what quality care means to them. In both cases, the questions could also reveal biases around gender and ethnicity as barriers to quality care. All participants were asked to provide sociodemographic data recorded using RedCap Software. All instruments were tested and adjusted during a pilot test in five health units in the State of Mexico.

Three researchers, a nutritionist, a psychologist, and a nurse, conducted the interviews and focus groups. Concerning the user interviews, the researcher directly requested the interview with the health personnel or the user at the medical office area. Due to time restrictions in the units, priority was given to conducting at least one interview with an HP and another with a user. In the case of the focus groups, these were convened by health center staff one day in advance, requesting five to six users belonging to the same stage of life (preconception, pregnancy, postpartum, infancy, or preschool) in the units at 8:00 a.m. However, due to difficulties in summoning or locating participants, only nine out of the expected 30 focus groups (five in each state) were conducted. In the State of Mexico, holding a focus group was impossible.

A total of 88 health units were visited. We conducted 87 interviews with health personnel, 85 with users, and nine focus groups were interviewed, in which 39 women in different stages of life participated (Table 1). All interviews and focus groups were audio-recorded and transcribed by seven field researchers.

**Table 1:** Key informants and data collection techniques

Place (n = health units)	Number and type of informants (n = they belong to an indigenous community)		Techniques
	Users	Health providers	
State of Mexico (*n* = 22)	**16 Users (*n*** **= 7)**:	**20 Health providers (*n*** **= 3)**:	
	• 15 Women interviewed • A Man interviewed	• 4 Physicians • 11 Nurses • 3 Nursing assistant • 2 Nutritionists	36 Interviews
Chihuahua (*n* = 11)	**23 Users (*n*** **= 4)**:	**12 Health providers (*n*** **= 2)**:	
	• 11 Women interviewed • 12 Women in focus group	• 4 Physicians • 5 Nurses • 2 Nutritionists • 2 Interpreter support	• 23 Interviews • 2 Focus group
Chiapas (*n* = 16)	**19 Users (*n*** **= 3)**:	**14 Health providers** (***n*** **= 3)**:	
	• 13 Women interviewed • A Man interviewed • 5 women in focus group	• 2 Physicians • 7 Nurses • 1 Nursing assistant • 4 Nutritionists	• 28 Interviews • 1 Focus group
Oaxaca (*n* = 16)	**31 Users (*n*** **= 4)**:	**11 Health providers (*n*** **= 3)**:	
	• 15 Women interviewed • 16 women in focus group	• 3 Physicians • 7 Nurses • 1 Nutritionist	• 26 Interviews • 2 Focus group
Veracruz (*n* = 18)	**24 Users (*n*** **= 11)**:	**18 Health providers (*n*** **= 4)**:	
	• 13 Women interviewed • 11 Women in focus group	• 12 Physicians • 5 Nurses • 1 Nutritionist	• 31 Interviews • 2 Focus group
Yucatan (*n* = 12)	**28 Users (*n*** **= 11)**:	**12 Health providers (*n*** **= 1)**:	
	• 15 Women interviewed • A Man interviewed • 12 women in focus group	• 1 Physician • 3 Nurses • 7 Nutritionist • 1 Social worker	• 28 Interviews • 2 Focus group

A subsample of 40% of the interviews (*n* = 66) and all nine focus groups were used for the analysis because a theoretical saturation was reached. A thematic analysis with a descriptive phenomenological approach was carried out to find patterns concerning the quality of care [25]. All the interviews were loaded into the free software Taguette to be coded following the codebook that included 25 deductive categories and five inductive [26, 27]. The deductive categories were functional to analyze the issues addressed directly by the instrument and that respond to the research objectives, such as the understanding of the quality of nutritional care and the care practices of health personnel. With the inductive categories, it was possible to systematize unexpected findings or topics, such as the concept of “treat well” or specific cases of malnutrition. A researcher conducted the coding, and subsequently, the results were discussed in sessions with two other researchers.

The study was approved by the Ethics Committee from the Universidad Iberoamericana (103/2021). The identity of the participants was kept anonymous, and permission to publish the results was obtained through informed consent.

## Results

The final sample included 95 health units. Table 2 shows the location of the evaluated units by state. Only two units in Oaxaca were not visited due to insecurity problems or difficulty accessing the sites. Of the health-assessed units, 34.7% were in rural areas and 65.3% in urban areas.

**Table 2:** Location of Primary Health Care units evaluated

	State						
	**Total** (*n* = 95)	Veracruz (*n* = 18)	Chiapas (*n* = 16)	Oaxaca (*n* = 16)	State of Mexico (*n* = 22)	Yucatan (*n* = 12)	Chihuahua (*n* = 11)
Area*							
*Rural*	33 (34.7%)	8 (44.4%)	5 (31.3%)	3 (18.8%)	9 (40.9%)	4 (33.3%)	4 (36.4%)
*Urban*	62 (65.3%)	10 (55.6%)	11 (68.8%)	13 (81.3%)	13 (59.1%)	8 (66.7%)	7 (63.6%)

* Rural areas are defined as localities with less than 2500 inhabitants, and urban areas are defined as localities with 2500 inhabitants or more

### Quality of maternal and child nutritional care

**Fig. 1:**
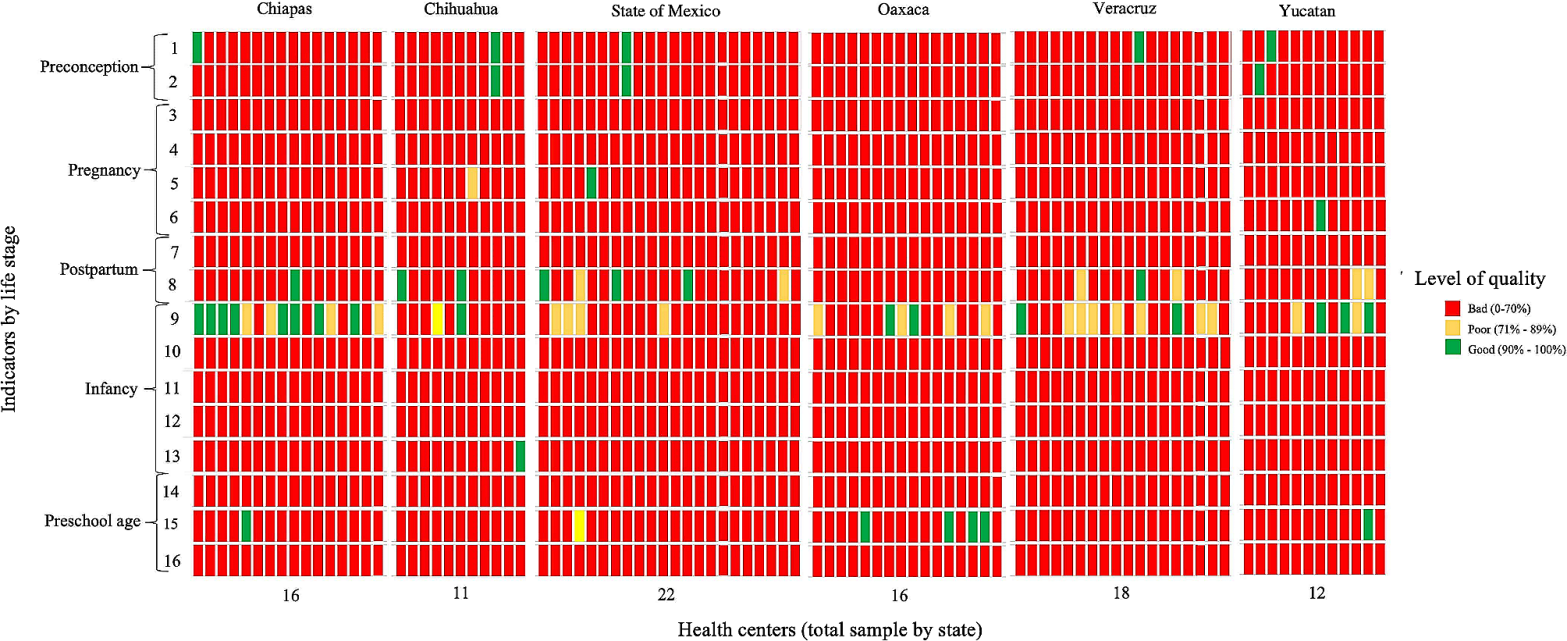
Quality of nutritional care by indicator, health unit and state. Mexico, 2020–2021. 1- Weight control strategies; 1- Folic acid supplementation; 3- Supplementation pregnancy; 4- Anemia screening; 5- Adequate follow-up; 6- Nutritional evaluation and vitamin supplementation in adolescent pregnancy; 7- Guidance on techniques for effective latching, breast massage and milk expression; 8- Guidance on postpartum weight control; 9- Promotion of exclusive breastfeeding, continued breastfeeding and complementary feeding; 10 - Assessment of nutritional status; 11 - Recommendation to reduce energy intake and fast food in infants with obesity; 12- Follow-up of patients with undernutrition; 13- Timely detection and identification of risk factors for iron deficiency anemia in patients under two years of age with undernutrition; 14- Physical activity and nutritional recommendations; 15- Preschool age children with anthropometric assessment; 16- Recommendations to reduce energy intake and fast food in preschool age children with obesity

Figure 1 presents the results per state of each indicator of nutritional care quality using the traffic lights system, providing an overview of maternal and child nutritional care quality at the PC, where each square represents an evaluated health unit. Our results show that maternal and child nutritional care quality in almost all the evaluated PCs is bad, evidenced by a high proportion of indicators per state marked in red (percentage of compliance < 70).

Despite these results, some indicators displayed higher compliance in several health units, such as “Promotion of exclusive breastfeeding, continued breastfeeding, and complementary feeding” (Indicator 9). Specifically in Chiapas, eight of the evaluated health units presented good quality for this indicator (percentage of compliance > 90). At the same time, four showed poor quality (percentage of compliance 70–90), and the other four had bad quality. However, on average, poor quality was obtained (percentage of compliance: 79.8). In general, this indicator obtained higher percentages of compliance in the states evaluated than the other indicators (Chihuahua: 30.7%; Oaxaca: 62.3%; Estado de México: 40.8%; Veracruz: 60.3%; Yucatan: 50.4%). No significant differences were found in the level of nutritional care quality when comparing rural and urban areas and ethnicity.

Regarding the other indicators, the results were variable in each state. However, we can assume a bad quality of nutritional care because red predominates across all indicators and states. Indicators “Weight control strategies” (Indicator 1) and “Folic acid supplementation” (Indicator 2) in the preconception period had percentages of compliance lower than 14% in all states, and it was identified a lack of clinical records corresponding to this life stage in all health units. From the indicators belonging to pregnancy stage, “Supplementation pregnancy” (Indicator 3) had the lowest compliance in all states (Chiapas: 0.0%; Chihuahua: 0.4%; Oaxaca: 0.0%; Estado de México: 0.0%; Veracruz: 0.0%; Yucatan: 0.0%; global 0.1%), while the indicator “Adequate follow-up” had the highest (Chiapas: 31.4%; Chihuahua: 27.7%; Oaxaca: 20.0%; Estado de México: 21.9%; Veracruz: 19.9%; Yucatan: 21.7%; global 23.8%). In the postpartum stage, the indicator “Guidance on techniques for effective latching, breast massage, and milk expression” (indicator 7) had a compliance of 0.0% in all states, except in Estado de México, which had a 0.3%. In the same life stage, the indicator “Guidance on postpartum weight control” (Indicator 8) had better percentages of compliance (Chiapas: 14.8%; Chihuahua: 18.2%; Oaxaca: 11.4%; Estado de México: 33.7%; Veracruz: 20.7%; Yucatan: 29.2%; global: 21.4%).

The indicator “Assessment of nutritional status” from the infancy stage (Indicator 10) obtained low percentages of compliance in all states; in Veracruz and Chiapas, the compliance was 0.0%, whereas in Oaxaca and Chihuahua, it was 0.4%, and in Yucatan and Estado de México 0.5% and 2.5%, respectively. The indicator “Recommendation to reduce energy intake and fast food in infants with obesity” from the infancy stage (Indicator 11) had a compliance of 0.0% in the six states. The indicator “Follow-up of patients with undernutrition” (Indicator 12) had a compliance of 0.0% in all states, except in Yucatan (6.4%). Regarding preschool age, the indicator “Physical activity and nutritional recommendations” (Indicator 14) had global compliance of 0.6%; Oaxaca, Yucatan, and Veracruz had a percentage of compliance of 0.0%, Estado de México had 0.6%, Chiapas 1.9%, and Chihuahua 0.8%.

**Fig. 2:**
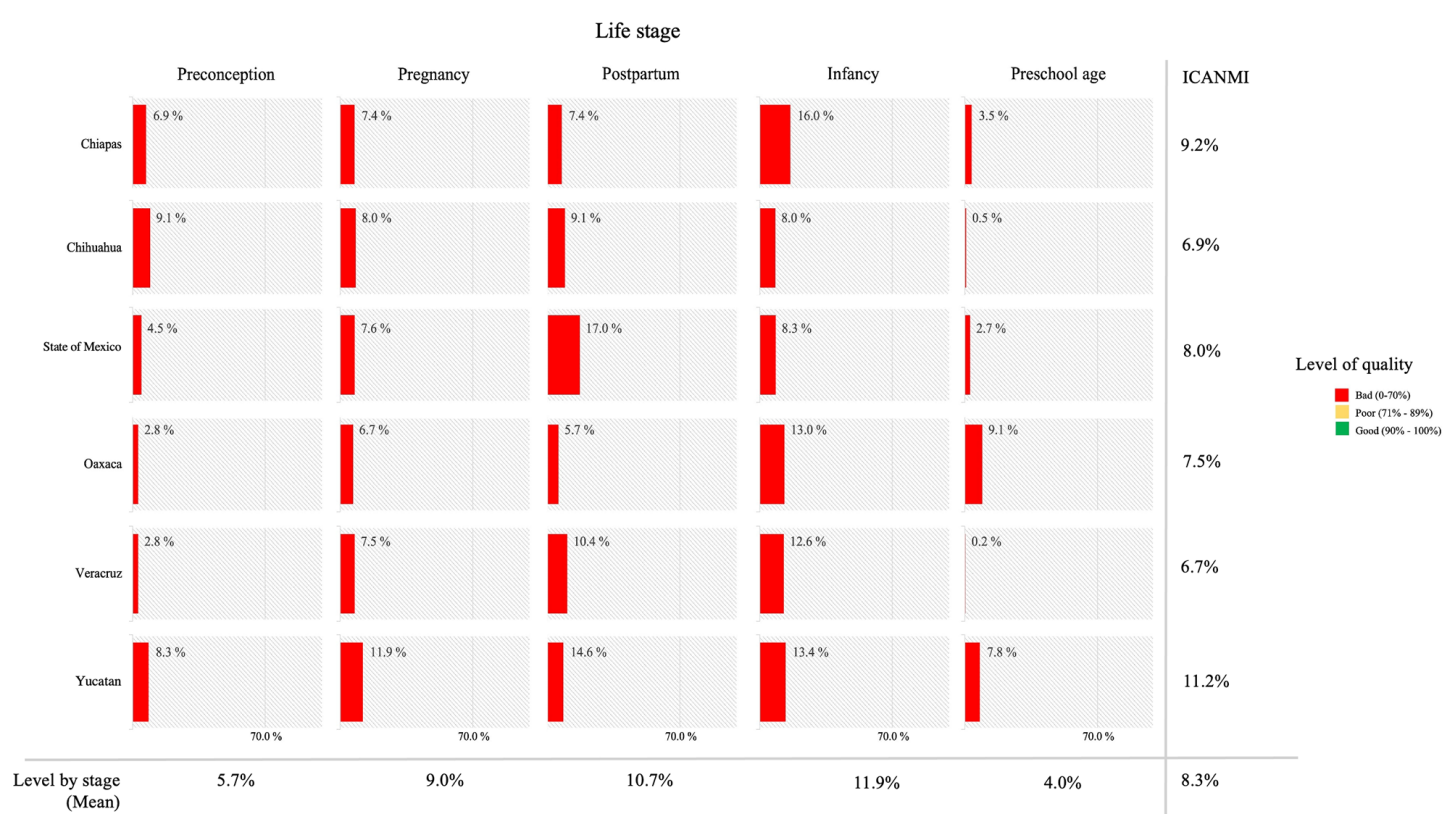
Quality of nutritional care during preconception, pregnancy, early childhood, and preschool age. Mexico, 2020–2021. ICANMI: Quality Index of Maternal and Child Nutritional Care

In Fig. 2, the results of stage indexes are shown by state. These indexes reflect the results mentioned above. The quality of nutritional care at PC during preconception, pregnancy, early childhood, and preschool age was bad in the six states. The preconception index had a global compliance of 5.7%, with the highest value in Chihuahua (9.1%) and the lowest in Oaxaca and Veracruz (2.8%). The pregnancy index had a global compliance of 9.0%, with the highest in Chiapas (12.3%) and the lowest in Oaxaca (6.7%). Concerning the postpartum index, Estado de México obtained the highest percentage of compliance (17.0%), and Oaxaca obtained the lowest (5.4%). Chiapas had better compliance in the Infancy index (16%); the lowest value was for Chihuahua (8.0%). Finally, the Preschool index had the highest compliance in Oaxaca (9.1%) and the lowest in Veracruz (0.2%). Finally, INCANMI was unfavorably the six states, beginning with Yucatan at 11.2%, followed by Chiapas at 9.2%, the State of Mexico at 8.0%, Oaxaca at 7.5%, Chihuahua at 6.9%, and Veracruz at 6.7%.

### Qualitative results

The main characteristics of the PHs and users are shown in Tables 3 and 4. According to health professionals and users, the most frequent diagnoses of malnutrition were anemia in pregnant women, mild malnutrition in childhood, and overweight and obesity in pregnant women and children under five years of age. The lack of financial resources is the main cause of malnutrition identified by health personnel and users, which makes it hard to follow nutritional recommendations or treatments. In addition, it was found that there needs to be a culture of health prevention or materials or strategies to promote it.

**Table 3:** Sociodemographic characteristics of health professionals

	Chihuahua (*n* = 13)	State of Mexico (*n* = 20)	Veracruz (*n* = 18)	Oaxaca (*n* = 11)	Chiapas (*n* = 14)	Yucatan (*n* = 12)	Total (*n* = 88)
**Age (years)**							
*media (DE)*	37.4 (7.4)	38.3 (9.6)	43.6 (8.8)	41.9 (9.4)	40.5 (7.4)	39.7 (16.1)	40.2 (9.9)
**Sex n(%)**							
Woman	10 (76.9)	16 (80)	15 (83.3)	9 (81.8)	9 (64.3)	6 (50)	65 (73.9)
Man	3 (23.1)	4 (20)	3 (16.7)	2(18.2)	5 (35.7)	6 (50)	23 (26.1)
**Ethnicity** ***n(%)***							
No	11 (84.6)	17 (85)	14 (77.8)	8 (72.7)	11 (78.6)	11 (91.7)	72 (81.8)
Yes	2 (15.4)	3 (15.0)	4 (22.2)	3 (27.3)	3 (21.4)	1 (8.3)	16 (18.2)
**Education** ***n(%)***							
Elementary school	1 (7.7)	0	0	0	0	0	1 (1.1)
Middle school	1 (7.7)	0	0	0	0	1 (8.3)	2 (2.3)
High school	0	3 (15)	1 (5.6)	0	0	2 (16.7)	6 (6.8)
Bachelor’s Degree	11 (84.6)	15 (75)	15 (83.3)	9 (81.8)	8 (57.2)	3 (25)	61 (69.3)
Technical Major	0	0	0	1 (9.1)	3 (21.4)	0	4 (6.6)
Post graduated	0	2 (10)	2 (11.1)	1 (9.1)	3 (21.4)	6 (50)	14 (15.9)
**Position** ***n(%)***							
Physician	4 (30.8)	4 (20)	12 (66.7)	3 (27.3)	2 (14.3)	1 (8.3)	26 (29.5)
Nurse	5 (38.5)	11 (55)	5 (27.8)	7 (63.6)	7 /50)	3 (25)	38 (43.2)
Auxiliary Nurse	0	3 (15)	0	0	1 (7.1)	0	4 (4.6)
Nutritionist	2 (15.4)	2 (10)	1 (5.6)	1 (9.1)	4 (28.6)	7 (58.3)	17 (19.3)
Social worker	0	0	0	0	0	1 (8.3)	1 (1.1)
No answer	2 (15.4)	0	0	0	0	0	2 (2.3)

Adapted from Hoyos-Loya E. et al. [1]

Hoyos-Loya E, Pérez Navarro C, Burrola-Méndez S, Hernández-Cordero S, Omaña-Guzmán I, Sachse Aguilera M and Ancira-Moreno M (2024) Barriers to promoting breastfeeding in primary health care in Mexico: a qualitative perspective. *Front. Nutr.* 10:1278280. doi: 10.3389/fnut.2023.1278280

**Table 4:** Sociodemographic characteristics of users

	Chihuahua (*n* = 22)	State of Mexico (*n* = 13)	Veracruz (*n* = 24)	Oaxaca (*n* = 16)	Chiapas (*n* = 19)	Yucatan (*n* = 30)	Total (*n* = 124)
**Age (years)**							
*Media (DE)*	28.2 (6.1)	25.5 (5.2)	28.8 (6.9)	29 (4.8)	29 (6.4)	27.3 (8.5)	28.1 (6.7)
**Sex** ***n(%)***							
Woman	22 (100)	12 (92.3)	24 (100)	16 (100)	18 (94.7)	29 (96.7)	121 (97.6)
Man	0	1 (7.7)	0	0	1 (5.3)	1 (3.3)	3 (2.4)
**Ethnicity** ***n(%)***							
No	18 (81.8)	6 (46.2)	13 (54.2)	12 (75.0)	16 (84.2)	19 (63.3)	84 (67.7)
Yes	4 (18.2)	7 (53.8)	11 (45.8)	4 (25.0)	3 (15.8)	11 (36.7)	40 (32.3)
**Civil status** ***n(%)***							
Single	2 (9.1)	0	3 (12.5)	1 (6.2)	0	2 (6.7)	8 (5.5)
Married	9 (40.9)	3 (23.1)	11 (45.8)	6 (37.5)	8(42.1)	18 (60)	55 (44.4)
Divorced	1 (4.6)	0	0	0	0	0	1 (0.8)
Common Law	10 (45.4)	10 (76.9)	9 (37.5)	9 (56.3)	11 (57.9)	10 (33.3)	59 (47.6)
Widow	0	0	1 (4.2)	0	0	0	1 (0.8)
**Number of children** ***n(%)***							
One	1 (4.6)	4 (30.8)	4 (16.7)	0	1 (5.3)	2 (6.7)	12 (9.7)
2 to 3	8 (36.4)	3 (23.1)	8 (33.3)	6 (37.5)	5 (26.3)	11 (36.7)	41 (33.1)
Up to 3	12 (54.5)	5 (38.5)	11 (45.8)	7 (43.8)	12 (63.2)	10 (33.3)	57 (45.9)
No answer	1 (4.5)	1 (7.7)	1 (4.2)	3 (18.8)	1 (5.2)	7 (23.3)	14 (11.3)
**Education** ***n(%)***							
None	1 (4.6)	0	0	0	0	0	1 (0.8)
Elementary school	5 (22.7)	2 (15.4)	4 (16.7)	3 (18.8)	1 (5.3)	5 (16.7)	20 (16.1)
Middle school	8 (36.4)	8 (61.5)	9 (37.5)	8 (50.0)	6 (31.6)	18 (60)	57 (46)
High school	3 (13.6)	3 (23.1)	8 (33.3)	3 (18.8)	6 (31.6)	7 (23.3)	30 (24.2)
Bachelor’s Degree	5 (22.7)	0	2 (8.3)	2 (12.5)	4 (21.2)	0	13 (10.5)
Technical Major	0	0	0	0	1 (5.3)	0	1 (0.8)
Post graduated	0	0	1 (4.2)	0	1 (5.3)	0	2 (1.6)

Adapted from Hoyos-Loya E. et al. [1]

Hoyos-Loya E, Pérez Navarro C, Burrola-Méndez S, Hernández-Cordero S, Omaña-Guzmán I, Sachse Aguilera M and Ancira-Moreno M (2024) Barriers to promoting breastfeeding in primary health care in Mexico: a qualitative perspective. *Front. Nutr.* 10:1278280. doi: 10.3389/fnut.2023.1278280

Out of the 88 health units evaluated, only 17 nutritionists were available for interviews, as this number represented the total count of nutritionists found in the visited health centers. In most units, these professionals were absent. Additionally, it was reported in three health centers that a nutritionist visited every two weeks to provide nutritional care. A significant barrier to their work is that the physician must refer the patient to a nutrition consultation. Their nutritional follow-up is almost nil since patients do not return for a second consultation. So, most of the diagnoses for children’s malnutrition are made by physicians, mainly based on the cut-off points of the WHO. If any form of malnutrition is detected, they issue general recommendations such as reducing fats and sugars and including fruits and vegetables in the daily diet. They recognize the lack of knowledge and training in this area, and some of them mentioned that during academic formation, they only received general information on healthy eating.

Although greater structural deficiencies were observed in rural health units, the staff and users in rural and urban centers reported shortages of equipment, medicine, personnel, and materials, indicating a systemic problem. Another structural barrier was the disappearance of the Prospera social cash transfer program, which forced the beneficiary to attend the health center monthly for check-ups for mothers and their children to receive financial support from the government. There is no co-responsibility to obtain the money, and users only go to health units when their children are sick.

In health units where users who identify as indigenous are cared for, they said they had never felt discriminated against because of their ethnic origin. However, health personnel from Chihuahua mentioned that a barrier to providing quality care was that they did not speak the indigenous language. In this state, two translators who support communication between doctor and patient were found in a clinic. In Yucatan, Chiapas, and the State of Mexico, the nursing staff acknowledged speaking a local indigenous language, favoring women’s care.

Users also do not perceive a gender bias in the medical attention they receive and think they are attended to well. On the other hand, health personnel consider that machismo and other practices of control over women persist in some localities, which can affect their attendance at control consultations or following nutritional recommendations. Some answers from the physicians reveal that there is no medical care from a gender perspective since they point to women as the only ones responsible for their children’s health or consider that sometimes they do not understand the medical indications and, therefore, must be explained to a man. Most of the users go to the health center alone because the men are the ones who work and do not have time to accompany them. The women do not see the above as a problem for them or the care of their children.

The concept of “treat well” was a continuum among all interviewees. For health personnel, “treating well” means greeting them, calling them by name, and answering their patients’ questions. For users, it implied that the physicians or nurses attended to them during consultations and had patience. In both cases, “treating well” was the basis of quality care; it was even the only thing they considered essential or expected to receive.

Table 5 shows some representative quotes from health personnel and users, organized thematically by the stages of nutritional care and understanding this concept.

**Table 5:** Perception of health personnel and users about nutritional quality care

Topic	Health Personnel	Users
**What is Quality of Nutritional Care?**	Quality is communication, communicating with the patient and giving her that confidence. If you give her that confidence, then you give her warmth. In this way, you are giving the patient that quality, the confidence of also being able to express themselves - Physician, urban health center, Oaxaca When the medical consultation is understandable; speaking to the patients in such a way that they understand, not telling the mother to buy such milk that is sold in such a supermarket, because sometimes they do not have the resources. In other words, putting myself at the level of the population. That would be quality care. Giving them what is within their reach, recommending, for example, the “yard diet”, the “milpa” diet; if there is fruit in your house, eat that; if there are animals in your house eat that. I think that would be quality care. And, obviously, don’t raise your voice, don’t scold, talk to her with respect - Nutritionist, urban health center, Yucatan	[Quality of Nutritional Attention] is that they can help me so that I can feed my children correctly and know what benefits we have when feeding them correctly - Mother of a preschool-age child, rural health center, State of Mexico I don’t know very well what it is… [maybe] that they explain to me what I should eat, how to feed my baby - Postpartum woman, urban health center, Veracruz
**Prevention**	There is a lot of general consultation and due to the lack of time in nursing, guidance is not given. Before, there was a social program in which we brought people together and used to show demonstrations a lot. But, since the pandemic, we no longer do those things. Now, sometimes I give a talk to those who are here outside [in the waiting room] waiting for a consultation; we make small groups of four mothers and there I am giving them nutrition training - Nutritionist, rural health center, Veracruz The nutrition service is not given here [in the infirmary], only a brief orientation or a talk about what the Eatwell Plate is. Mainly, they are given information to try avoiding the consumption of dairy foods, fats, meats, sugars, soft drinks, and sweetened water, eating more fruits, vegetables, natural water, and exercising at least 15 min a day. The only nutrients [supplements] they are given are polyvitamins, which are the only ones we have here, and also some folic acid. - Nurse, urban health center, State of Mexico	I don’t bring my son frequently mainly because… Well, I don’t know. Maybe we don’t have that culture of keeping a medical check-up and due to the lack of information; we don’t know if they give us that kind of care here [nutritional care]- Mother of a child in preschool age, urban health center, Oaxaca Since before I got married, I began to prepare my body. I started coming to consultations so that the doctor would tell me the processes that I had to follow to be able to get pregnant, to plan a pregnancy. I took that control before and during the pregnancy. And after the pregnancy, I continued with the necessary vitamins. The doctor told us that breast milk is essential for the baby and I followed her suggestion to give him breast milk until he was 2 years old. Now I do the same thing, I prepare my body before I get pregnant for the second time. - User in the preconception stage, focus group at a rural health center, Veracruz
**Diagnosis and treatment**	To diagnose overweight in pregnant women we rely on the BMI; I do not have a specific table for pregnant women, I use the regular one, that is, as if she were not pregnant. They are always invited to reduce the levels [consumption] of certain foods, such as flour, tortillas, bread, soft drinks, and sugary foods, in addition to walking half an hour, and it is explained to them that pregnancy does not mean that they are not going to exercise, they could start walking - Physician, rural health center, Chihuahua A barrier to treatment is that there are almost no supplements, vitamins, or B complex. Sometimes there are some, and others we run out of them, seasonally. There are families that do not have enough money for the daily consumption of vegetables or protein. Protein is very scarce here, maybe there are families that eat chicken every fortnight or eat meat once a month. So, that also limits them a lot and there are seasons in which we can help them or give them vitamins, but when there are none, we cannot do anything - Nutritionist, urban health center, State of Mexico	Before I got pregnant I was diagnosed with anemia. When I came to my consultation I told the doctor and he told me to eat more foods rich in iron, such as beans, and lentils, and that I had to keep myself fed more so that my baby would grow healthy (…) but they have not clearly stated what healthy foods I should eat and what I shouldn’t - Pregnant woman, urban health center, Oaxaca I have tried to eat well, but the prices of good food or good ingredients are a bit high and salaries are low here, so that forces us to eat whatever. I have tried to buy broccoli, romaine lettuce, vegetables, and fruits. In other words, cooking a healthy broth is a bit expensive compared to, for example, making a scrambled egg and tortilla to eat. And if we want to accompany the food with a natural drink, so as not to drink soda, it is even more expensive - Mother of a child in the preschool stage, urban health center, Yucatan
**Barriers to quality care**	An important barrier is the interconsultation, the patients do not come with me because they have to see me after going to the physician and they do not want or cannot stay that long (…) I need space, it is very small and due to the weather we cannot close the door and we need privacy with the patient. I would like to have a baby scale, to corroborate the data taken by the nurse, and also some simple devices such as a glucometer, a protein meter, a caliper, but I think they are not provided in our health system—Nutritionist, urban health center, Chiapas The time we have for each consultation is 15 to 20 min. I believe that this affects negatively because finally we are facing a population that needs a lot of education and, for example, I can realize that the patient has many doubts or has not understood everything, but I can no longer dedicate more time to it. So I think that this is inadequate attention, not because I want to do it that way, but because that’s the way the system is—Physician, rural health center, State of Mexico	More doctors are needed so that they can care for us well, because sometimes we come and there are no doctors or they are unemployed. And I don’t know if there is already a nutritionist, but it is necessary. There is also no night shift and when there is an emergency and it is late there is no one to provide service, in fact there are no ambulances… well, there are no medicines either—Mother of child in the infant stage, urban health center, Oaxaca When I come to see my child and they prescribe medication, I have to buy it. In fact, throughout my pregnancy when blood tests were needed, there was never any laboratory staff to do them and I had to pay for them elsewhere, I also had to buy other medicines or supplements, the only thing they gave me was folic acid—Mother of a child in infant stage, urban health center, Chiapas
**Facilitators to quality care**	The authorities are always giving us material to work with, I had never had food replicas (for nutritional education) and here they give them to us. There is also good communication with the staff, I have a lot of support for promotion, the nursing staff help me so that the patient does not run away or they notify me when children are scheduled for vaccinations and while the mothers wait we go and give them a talk about nutrition. There really is teamwork and we are all coordinated—Nutritionist, urban health center, Yucatan For community workshops we have flipcharts, we as a nurses also prepare informative wall newspapers and we have the DVD in Mazahua on women’s health to project it in the talks with them, which have had an important impact.—Nurse, rural center health, State of Mexico	The health center has a special day for pregnant women, so they call us on that day and if we arrive early they see us first. The health center opens at 8 am, they put us on a list to be seen in that order. They take us to the nurse, who takes our vital signs and blood pressure, she weighs and measures us, and then we go to the physician. The attention of the health personnel is good—Pregnant woman, rural health center, State of Mexico The waiting time depends on how many patients there are. We only wait for how long it takes for the doctor or nutritionist to give the consultation to the patient who is before you, but it is not more than 15 or 20 min because while they are treating the other person, the nurse measures you, takes your weight, pressure and hear the baby’s heartbeat.—Pregnant woman, rural health center, Veracruz (Focus group)
**Gender perspective**	Here the common thing is that the man is the one who works, so he only sends the wife to the consultation. They only accompany them if they are from another community, but in general, for them coming to the health center is a waste of time—Nurse, rural health center, Chiapas. The woman is a little reserved. We have to be empathetic to be able to explain things to her and accept her recommendations, because if her husband doesn’t give her permission, I can’t give her guidance on issues like family planning. If the husband says “I don’t want my wife to take contraceptives”, she obeys him and she can have 6–7 children. Normally the mother is the one who is always looking after the children and the father just says: “I work, I bring the money, I don’t care about the rest” (…) I have heard patients say: “It’s that my husband doesn’t agree with me coming to the health center every month, he says that I’m just here to waste time because my son is fine,” and they no longer come to check on the child—Nurse, urban health center, Veracruz	I accompany my wife to the consultation because we both care about the health of the children and also about various situations that may occur, between the two of us we provide support, we support each other, especially when the diagnosis they can give us is not good (… ) I consider that it is very important to come to explain to the doctor the symptoms that the child presents, sometimes the spouse can miss some detail or medication that we gave her or something that we could observe in the child that she perhaps did not, and that’s how it is. we are both here to give each other that support—Father of a child in preschool stage, urban health center, Oaxaca. My mother or my partner comes with me. (I prefer to come) Accompanied, because many times I don’t understand much of what they are saying to me, and maybe my companion did understand well or so.—Pregnant women, rural health center, Chihuahua (Focus Group) From time to time my husband comes with me, because he works. (I prefer to come) By myself. I don’t know, it’s like when he’s here I don’t… I hardly even speak, because he’s just looking for the moment to criticize me, to laugh, it’s so… [LAUGHS] “and did you notice what you said?” he tells me, and I am there with all the shame in the world. It does not leave me well in front of the physician—Pregnant women, rural health center, Chihuahua (Focus Group) I always come alone, it’s fine, I’m used to it and my husband works. But sometimes I would like him to come, for him to hear that the physician recommends that I take care of myself, so that he doesn’t think that I’m not lying when I said that I have to rest or something like that—Pregnant women, rural health center, Chihuahua (Focus Group)
**Intercultural aspects**	The problem is all the localities that we serve here and well, quite a few indigenous children arrive with low weight or with an advanced degree of malnutrition, and well… sometimes the indigenous are inexpressive, and although I don’t have that much experience with the indigenous community, I feel that for them it is the same if their child is sick or healthy, I think they are not so interested in health. 98 or 99% of children with malnutrition are indigenous and I feel that there is a lack of awareness on the part of the government towards this community. There are indigenous people who are more civilized, but here we have indigenous people who are practically like little animals, they live in caves and things like that… it sounds ugly, but that’s the way it is—Physician, Community general Hospital, Chihuahua I worked in the cleaning area and saw how the Tarahumara population arrived with their children. Sometimes the parents worried because the doctor did not understand them, and they were sad because they did not know what to do. So that to me, well, it kind of hurt me, and I said ‘why don’t the physicians tell me to ask the parents what the child has, since when did he get sick, how many days has he been like this?‘, all of that. But once a physician did ask me: ‘Hey, how do we support this child who arrived and the relatives don’t understand me?‘, and then I told him: ‘I’ll help you, doctor’, and I already translated for him. I felt so glad to help them! And that’s where my work as an interpreter for the Tarahumaras began - Interpreter, Integrated Community Hospital, Chihuahua	In Spanish they speak differently than Mayan, if there was someone who speaks Mayan here and explains everything about nutrition, we would understand. It has been difficult to understand, for example, how you can prevent losing your baby or what you should do when the baby is coming down, if you should come here or to another place, that information is what I don’t understand. Here they have not given me any material such as brochures or books on food in the Mayan language—Pregnant woman, rural health center, Yucatan

## Discussion

The quality of maternal and child nutritional care in PC in the states of Oaxaca, Yucatan, Chiapas, Chihuahua, State of Mexico, and Veracruz was bad between 2020 and 2021. This bad quality was identified for the nutritional care in all the studied life stages (preconception, pregnancy, postpartum, infancy, and preschool age), indicating the urgent call for action to improve the quality of maternal and child nutritional care in health units of PC.

Our results are alarming, given the long-lasting adverse effects of malnutrition at different life stages, particularly early in life (first 1,000 days and early childhood), for the health and development of both individuals and society [28]. There is evidence showing that health services interventions to promote and support maternal and early-life nutrition are a unique opportunity “to prevent and treat malnutrition in all its forms” in the first 1,000 days of life and early childhood [29].

In Mexico, administrative regulations called “Official Mexican Norms” (NOMs) establish rules, guidelines, specifications, and minimum requirements applicable to a product, process, or service. Among these NOMs, some regulations of high technical specificity are established to comply with the regulations and laws in force. Specifically, there is a NOM for maternal and child health and nutritional care [30]. The indicators used to evaluate maternal and child nutritional care quality consider compliance with current Mexican and international recommendations and regulations. Hence, our results highlight that neither the international recommendations nor the current local regulations are being complied with; this is a missed opportunity for the Mexican health system to intervene at a preventive level.

From a qualitative perspective, there are structural limitations to providing quality nutritional care, ranging from the need for more equipment and supplies to the low presence of nutritionists in health centers. These factors are the first barrier to compliance with the NOMs. However, users also have contextual limitations to follow nutritional recommendations, such as economy and resistance due to uses and customs. Likewise, it was observed that the training of PHs focuses more on curative care than preventive care. However, since it was not the subject of study in this article, it is suggested that future research or studies may explore this aspect further.

The lack of adequate training for HP directly impacts the quality of health care in monitoring and treating various forms of maternal and child malnutrition. This issue has been substantiated by some studies [31, 32], highlighting the inadequacy in the knowledge and practices of first-contact HP to address infant malnutrition effectively since their professional training. Furthermore, it has been observed that HP tends to underestimate the prevalence of infant malnutrition and overestimate the frequency of detection practices [18].

During health emergencies such as the COVID-19 pandemic, primary care services are affected as all resources are directed towards secondary and tertiary levels of care. This situation could explain the reported shortage of medications and supplements during the study period. This scenario underscores the need to strengthen primary care services by establishing monitoring mechanisms that ensure a continuous supply of supplies to the units, even after overcoming the COVID-19 crisis [33].

Few studies have examined the circumstances under which health services are provided, contributing to ensuring that they meet the nutritional needs of users satisfactorily. To our knowledge, this is the first time that the quality of maternal and child nutritional care is evaluated in PC from preconception to preschool age.

The main strengths of this study are the robust methodology followed for the development and validation of the indicators and its gender equity and interculturality perspective [21]. The evidence generated in this work would help the Mexican Government and other countries in similar contexts strengthen their health systems and ensure high-quality nutritional care during preconception, pregnancy, the postpartum period, early infancy, and preschool age.

There is a detected need to focus on resolving infrastructure deficiencies (equipment, medicine, materials) and the limited hiring of nutritionists in both urban and rural units. Furthermore, nutritional training for HP, especially physicians, is highly necessary since they make most of the nutritional diagnoses and treatments in PC. It is important to improve their knowledge, attitudes, and practices to offer an effective nutritional care provision, if necessary, or motivate the referral to a nutritionist in the unit [34–36]. These measures not only contribute to optimizing the care provided but also have positive impacts on the overall health of patients.

It is necessary to mention some limitations. This study took place during the COVID-19 pandemic. It is important to consider that the evaluation and time frame had special characteristics caused by the COVID-19 pandemic and the public health measures for its containment. To respond to the emergency, health systems had to reorganize their resources, which could cause the neglect or closure of essential health services offered at the PC to the maternal and child population, one of the most vulnerable groups that are affected by this situation [37]. However, during the visit to the health centers, we identified that all the health workers had already returned to their usual work activities and assignment of functions. Another limitation was the quality of the registration information in the medical record.

## Conclusions

The quality of nutritional care in preconception, pregnancy, childhood, and preschool was bad in Chihuahua, State of Mexico, Veracruz, Oaxaca, Chiapas, and Yucatan.

The procedures for diagnosing and treating various forms of malnutrition are not followed per clinical practice guidelines, highlighting the need to provide ongoing training to HP in the field of nutrition. Additionally, units face various barriers that hinder the proper provision of health services, such as a shortage of equipment, medications, materials, and even insufficient HP.

These results call for urgent action to implement strategies to improve nutritional care quality at the PC in Mexico. It is urgent that the government and health authorities, together with other stakeholders, design, implement and evaluate actions aimed at improving the quality of nutritional care to help reduce the prevalence and incidence of the different forms of malnutrition during the stages of life evaluated in this work. This will impact not only maternal and child health and nutrition in the short term but also across generations in such a way that these actions at the PC could contribute to increasing productivity and prosperity in Mexico.

### Electronic supplementary material

Below is the link to the electronic supplementary material.

Supplementary Material 1

## Data Availability

The datasets used and/or analyzed during the current study are available from the corresponding author on reasonable request.

## Abbreviations

ANSI: American National Standards Institute
ICANMI: Quality Index of Maternal and Child Nutritional Care
LQAS: Lot Quality Acceptance Sampling
NOMs: Official Mexican Norms
PC: Primary Care
WHO: World Health Organization
