# Supplementary material for: Low quality of maternal and child nutritional care at the primary care in Mexico: an urgent call to action for policymakers and stakeholders
Source: Int J Equity Health. 2024 Feb 22;23:35. doi: 10.1186/s12939-024-02129-z (PMC10885649; doi:10.1186/s12939-024-02129-z)
Supplement: Supplementary file 1 — Supplementary Material 1 [file 12939_2024_2129_MOESM1_ESM.docx]

**Supplementary table 1. Assignment of samples by stratum and state**

| **Stratum** | **Size of medical unit*** | **Chiapas** | **State of Mexico** | **Oaxaca** | **Chihuahua** | **Veracruz** | **Yucatan** | **Total** |
| --- | --- | --- | --- | --- | --- | --- | --- | --- |
| 1 | Small | 3 | 3 | 2 | 3 | 3 | 1 |  |
|  | Medium | 1 | 3 | 1 | 2 | 1 | 1 |  |
|  | Large | 0 | 1 | 1 | 1 | 1 | 0 |  |
|  | **Total** | **4** | **7** | **4** | **6** | **5** | **2** | **28** |
| 2 | Small | 1 | 2 | 2 | 1 | 2 | 1 |  |
|  | Medium | 1 | 3 | 2 | 1 | 2 | 1 |  |
|  | Large | 3 | 4 | 2 | 2 | 3 | 2 |  |
|  | **Total** | **5** | **9** | **6** | **4** | **7** | **4** | **35** |
| 3 | Small | 3 | 1 | 3 | 0 | 2 | 2 |  |
|  | Medium | 1 | 1 | 1 | 0 | 1 | 1 |  |
|  | Large | 1 | 1 | 0 | 0 | 0 | 0 |  |
|  | **Total** | **5** | **3** | **4** | **0** | **3** | **3** | **18** |
| 4 | Small | 1 | 1 | 2 | 0 | 1 | 1 |  |
|  | Medium | 1 | 1 | 1 | 0 | 1 | 1 |  |
|  | Large | 1 | 1 | 1 | 0 | 1 | 1 |  |
|  | **Total** | **3** | **3** | **4** | **0** | **3** | **3** | **16** |

Stratum 1: health units located in non-indigenous rural areas.

Stratum 2: health units located in non-indigenous urban areas.

Stratum 3: health units located in indigenous rural areas.

Stratum 4: health units located in indigenous urban areas.

^*^ Small size: 1 general medicine office; medium size: 2 general medicine offices; large size: 3 or more general medicine offices.

**Supplementary table 2. Indicators to assess the quality of maternal and child nutritional care at Primary Health Care**

| **Life stage** | **Indicators and sub-indicators** | **Description** | **Formula** |
| --- | --- | --- | --- |
| Preconception | 1. **Weight control strategies**    1. Dietary recommendations    2. Physical activity/exercise recommendations    3. Pharmacological treatment recommendations    4. Surgical recommendations | Percentage of patients who have been prescribed, dietary, exercise, medical or surgical strategies for weight management three months before their pregnancy. | $\frac{\begin{aligned} The number of patients during the preconception period who have \\ been prescribed dietary, exercise, medical, or surgical strategies for \\ weight management three months before their pregnancy. \end{aligned}}{Total number of patients during the preconception period.}x100$ |
|  | 1. **Folic acid supplementation** | Percentage of patients who are prescribed folic acid three months before their pregnancy | $\frac{\begin{aligned} Number of patients in the preconception stage who were prescribed \\ folic acid supplementation in the three months prior to their pregnancy \end{aligned}}{Total number of patients during the preconception period} x100$ |
| Pregnancy | 1. **Supplementation in pregnancy**    1. Folic acid    2. Vitamin D | Percentage of patients with folic acid supplementation during the first trimester of pregnancy | $\frac{\begin{aligned} Number of pregnant patients who were prescribed folic \\ acid supplementation during the first trimester of pregnancy. \end{aligned}}{Total number of pregnant patients} x100$ |
|  | 1. **Anemia screening**     1. Blood biometry request in the first consultation    2. Blood biometry request around 25 weeks of pregnancy | Percentage of pregnant patients who have screened for anemia | $\frac{\begin{aligned} The number of pregnant patients to whom screening for anemia was \\ provided during the first pregnancy consultation and around 28 \\ gestational weeks \end{aligned}}{Total number of pregnant patients} x100$ |
|  | 1. **Adequate follow-up**    1. Adequate number of medical consultations    2. Recorded the patients weight at each visit | Percentage of patients with adequate prenatal follow-up. | $\frac{The number of patients with adequate prenatal follow-up.}{Total number of pregnant patients} x100$ |
|  | 1. **Nutritional evaluation and vitamin supplementation in adolescent pregnancy**    1. Nutritional diagnosis    2. Folic acid and iron prescription | Percentage of adolescent pregnant patients with nutritional evaluation and adequate vitamin supplementation. | $\frac{\begin{aligned} The number of adolescents pregnant with nutritional evaluation and \\ adequate vitamin supplementation. \end{aligned}}{Total number of adolescent pregnant patients.} x100$ |
| Postpartum | 1. **Guidance on techniques for effective latching, breast massage and milk expression** | Percentage of patients during the breastfeeding period who had guidance on techniques for effective latching, breast massage, and milk expression. | $\frac{\begin{aligned} The number of patients during the breastfeeding period with \\ guidance on techniques for effective latching, breast massage, \\ and milk expression. \end{aligned}}{Total number of patients during the breastfeeding period.} x100$ |
|  | 1. **Guidance on postpartum weight control** | Percentage of patients during the postpartum period with counseling about weight control. | $\frac{\begin{aligned} The number of patients during the postpartum period who received \\ counseling about weight control. \end{aligned}}{Total number of patients during the postpartum period.} x100$ |
| Infancy | 1. **complementary feeding.**    1. Promotion of exclusive breastfeeding in children under 6 months    2. Promotion of exclusive breastfeeding in children after 6 months and up to 2 years | Percentage of patients to whom their mothers had a promotion of exclusive breastfeeding, continued breastfeeding, and complementary feeding. | $\frac{\begin{aligned} The number of patients under two years old to whom their mothers have \\ a promotion of exclusive breastfeeding, continued breastfeeding, \\ and complementary feeding. \end{aligned}}{Total number of patients under two years old.} x100$ |
|  | 1. **Assessment of nutritional status**   10.1. Dietary history  10.2. Social and economic history  10.3 Nutritional diagnosis | Percentage of infants who had an assessment of nutritional status. | $\frac{The number of infants with an assessment of nutritional status.}{Total number of infants.} x100$ |
|  | 1. **Recommendation to reduce energy intake and fast food in infants with obesity** | Percentage of patients under two to five years old with obesity who have received recommendations to reduce energy and fast-food intake. | $\frac{\begin{aligned} The number of infants with obesity who recieved \mathrm{recommendations} \\ to reduce energy and fast-food intake . \end{aligned}}{Total number of patients under two years with obesity} x100$ |
|  | 1. **Follow-up of patients with undernutrition** | Percentage of patients with malnutrition who had adequate follow-up. | $\frac{\begin{aligned} The number of patients under two years old with malnutrition who \\ received adequate follow-up. \end{aligned}}{Total number of patients under two years with malnutrition.} x100$ |
|  | 1. **Timely detection and identification of risk factors for iron deficiency anemia in patients under two years of age with undernutrition** | Percentage of patients under two years old with malnutrition who had timely detection and identification of risk factors for the development of iron deficiency anemia. | $\frac{\begin{aligned} The number of patients under two years \\ old with malnutrition who had timely \\ detection and identification of risk factors for the development \\ of iron deficiency anemia. \end{aligned}}{Total number of patients under two years with malnutrition.} x100$ |
| Preschool age | 1. **Physical activity and nutritional recommendations**    1. Physical Activity/Exercise recommendations    2. Nutritional recommendations | Percentage of patients two to five years old who received physical activity and nutritional recommendations. | $\frac{\begin{aligned} The number of patients two to five years old who received physical \\ activity and nutritional recommendations \end{aligned}}{Total number of patients from two to five years old.} x100$ |
|  | 1. **Preschool age children with anthropometric assessment** | Percentage of patients two to five years old who had an anthropometric assessment on each consultation | $\frac{\begin{aligned} The number of patients two to five years old who had an \\ anthropometric assessment on each consultation. \end{aligned}}{Total number of patients from two to five years old.} x100$ |
|  | 1. **Recommendations to reduced energy intake and fast food in preschool age children with obesity** | Percentage of patients aged 2 to 5 years with obesity who received recommendations to reduce total energy intake and fast food consumption | $\frac{\begin{aligned} Number of patients aged 2 to 5 with obesity \\ who received recommendations to reduce total energy \\ intake and fast food consumption \end{aligned}}{Total number of patients from two to five years old with obesity.} x100$ |

Adapted from Ancira-Moreno M. et al, 2022 [1]

**References**

1. Ancira-Moreno M, Omaña-Guzmán I, Bautista-Morales AC, Acosta-Ruiz O, Hernández Cordero S, Burrola-Méndez S, et al. Development and validation of a new set of indicators to assess the quality of maternal and child nutritional care at the primary care. Front Med (Lausanne). 2022 Dec 7;9:3571.
